# Impaired glucose tolerance in healthy men with low body weight

**DOI:** 10.1186/1475-2891-10-16

**Published:** 2011-02-07

**Authors:** Kamila Jauch-Chara, André Schmoller, Kerstin M Oltmanns

**Affiliations:** 1 Department of Psychiatry and Psychotherapy, University of Luebeck, Luebeck, Germany

## Abstract

**Background:**

Impaired glucose tolerance (IGT) and high body mass index (BMI) are recognized risk factors for type 2 diabetes mellitus (T2DM). However, data suggest that also underweight predisposes people to develop T2DM. Here, we experimentally tested if already moderate underweight is associated with impaired glucose tolerance as compared to normal weight controls. Obese subjects were included as additional reference group.

**Method:**

We included three groups of low weight, normal weight, and obese subjects comprising 15 healthy male participants each. All participants underwent a standardized hyperinsulinemic-euglycemic glucose clamp intervention to determine glucose tolerance. In addition, insulin sensitivity index (ISI) was calculated by established equation.

****Results**:**

ISI values were higher in low and normal weight than in obese subjects (*P *< 0.010) without any difference between low and normal weight groups (*P *= 0.303). Comparable to obese participants (P = 0.178), glucose tolerance was found decreased in low weight as compared with normal weight subjects (*P = *0.007). Pearson's correlation analysis revealed a positive relationship between glucose tolerance and BMI in low (*P *= 0.043) and normal weight subjects (*P *= 0.021), an effect that was found inverse in obese participants (*P *= 0.028).

**Conclusion:**

Our study demonstrates that not only obese but also healthy people with moderate underweight display glucose intolerance. It is therefore suggested that all deviations from normal BMI may be accompanied by an increased risk of developing T2DM in later life indicating that the maintenance of body weight within the normal range has first priority in the prevention of this disease.

## Background

Type 2 diabetes mellitus is a global epidemic. A considerable body of evidence indicates that impaired glucose tolerance and body mass index represent risk factors for developing T2DM. To date, the association between BMI and impaired glucose tolerance is well documented in case of overweight and obesity [1] but little is known about the relationship between low weight and IGT. However, studies show a linear relationship between low birth weight and the prevalence of T2DM in adult life [2]. Moreover, IGT has been observed in severe malnutrition [3], certain forms of cancer [4], and anorexia nervosa [5], i.e. diseases accompanied by extremely low BMI within the scope of a pathologic state. These findings indicate that, apart from obesity, also a BMI < 20 kg/m^2 ^may be associated with IGT and therefore with an increased risk of T2DM.

Against this background, we hypothesized that, in analogy to obese subjects, also healthy individuals with moderate underweight display IGT. In order to test this hypothesis, we investigated whether glucose disposal rates, giving key information about glucose tolerance, and calculated insulin sensitivity by insulin sensitivity index (ISI) differ between low weight, normal weight, and obese healthy young men during a glucose clamp experiment which is considered as 'gold standard' method in this context in diabetes research [6]. A hyperinsulinemic-euglycemic clamp places plasma glucose concentration under the investigator's control and thus breaks the endogenous glucose-insulin feedback loop. The technique consists of an insulin infusion at predetermined fixed dosage and a variable glucose infusion rate. Under steady-state conditions of euglycemia, the glucose infusion rate equals glucose uptake by all tissues in the body [6] and is therefore a measure of glucose tolerance [7].

## Methods

We studied 15 low weight (BMI between 17.6 and 19.8 kg/m^2^; mean 18.97 ± 0.20 kg/m^2^), 15 normal weight (BMI between 20.2 and 23.5 kg/m^2^; mean 21.86 ± 0.30 kg/m^2^), and 15 obese (BMI between 30.1 and 43.6 kg/m^2^; mean 33.81 ± 1.10 kg/m^2^) male volunteers aged 18-30 years. All subjects had a regular sleep-wake cycle during the week before the experiments. Exclusion criteria were acute or chronic illness, diabetes in 1-degree family members, alcohol or drug abuse, smoking, competitive sports, shift work, exceptional physical or mental stress, and any kind of current medication. The study conformed to the Declaration of Helsinki and was approved by the Ethics Committee on Research Involving Humans of the University of Luebeck. All subjects gave written informed consent.

After an overnight fast of 12 hours, subjects arrived at the research unit at 8:00 a.m. One cannula was inserted into a peripheral vein on the back of the hand and a second cannula into an antecubital vein of the contralateral arm. The first blood sample was taken at 8:30 a.m. to determine baseline concentrations of circulating glucose and insulin. At 8:40 a.m., the glucose clamp was started by administration of an insulin bolus (H-insulin, Hoechst, Frankfurt, Germany) of 5 mU/kg body weight (BW)/min over 2 min. Thereafter, insulin was infused at a constant rate of 1.5 mU/kgBW/min until the end of the clamp. A 20% glucose solution was infused simultaneously at a variable rate to maintain plasma glucose values at approximately 5.5 mmol/l for 45 minutes. Thereafter, insulin infusion was stopped, and plasma glucose concentrations were normalized by increasing glucose infusion rates.

Blood samples were drawn at 5-minute intervals to measure plasma glucose concentrations (B-Glucose-Data-Management, HemoCue GmbH, Grossostheim, Germany) and every 10 minutes for the determination of hormones. Blood was centrifuged within 5 min after withdrawal and serum as well as plasma was kept at -72°C until assay.

Plasma glucose was measured by using the hexokinase method (Nichols Institute Diagnostics, Bad Vilbel, Germany) with coefficient of variation (CV) smaller than 4.2%. Serum insulin concentrations were measured by commercial enzyme-linked immunoassay (Immulite, DPC, Los Angeles, USA) with intra-assay CV < 5.2% and inter-assay CV < 6.1%.

Values are presented as mean ± SEM. To test for the criteria required for multivariate analyses, we performed a Kolmogorov-Smirnov test on each variable. A Kolmogorov-Smirnov *P*-value of .05 or greater was considered as a normal distribution All continuous variables were found normally distributed in each group (all Kolmogorov-Smirnov *Z *> 0.432, all *P *> 0.500). Thus, statistical analyses were based on analysis of variance for repeated measurements (ANOVA) with the factors "time" (for repeated measurements during the experiment) and "group" (for differences between normal, low weight, and obese participants). For pairwise comparisons between groups, unpaired Student's t-test was used. ISI was calculated as described by Eriksson et al. [8] and respective *P*-values were revealed by one-way ANOVA. Correlation analyses were performed by bivariate correlation analysis according to Pearson. A *P*-value less than 0.05 was considered significant.

## Results

**Figure 1:**
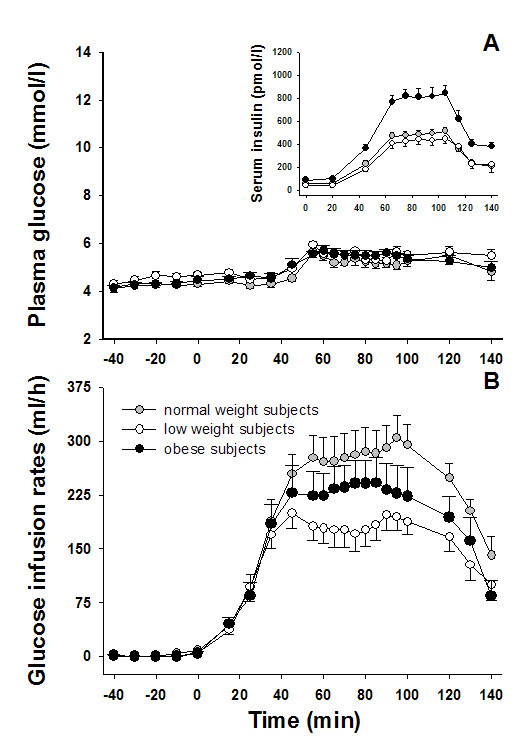
**Plasma glucose, glucose infusion rates, and insulin concentrations**. Mean ± SEM concentrations of plasma glucose (A), glucose infusion rates (B), and serum insulin (smart insert in Fig. 1A) during hyperinsulinemic-euglycemic clamp experiments in groups of low weight (open cycles), normal weight (gray cycles), and obese (black cycles) men (n = 15 per group).

**Figure 2:**
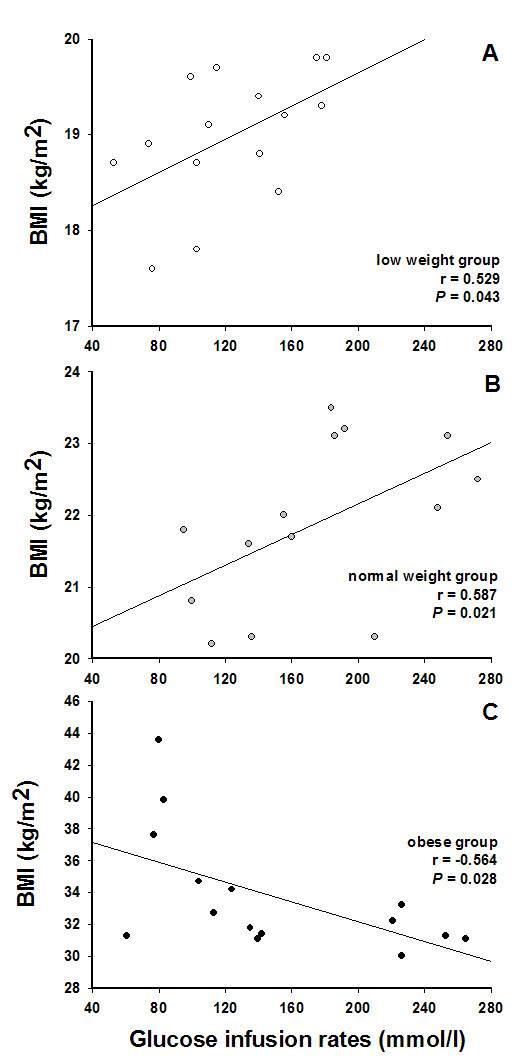
**Correlation between glucose infusion rates and BMI**. Correlation analyses between glucose infusion rates and BMI in low (open cycle; n = 15; Fig. 2A) and normal weight subjects (gray cycles; n = 15; Fig. 2B) as well as in obese men (black cycles; n = 15; Fig. 2C). Data show a positive correlation between BMI and glucose tolerance in low and normal weight participants which was found inverse in obese subjects.

As shown in Figure 1A, plasma glucose concentrations did not differ between low weight, normal weight, and obese subjects throughout the experiments (all *P *> 0.114 for group effect; all *P *> 0.409 for group by time interaction). Glucose infusion rates required to maintain target glucose values during the clamp were significantly decreased in low as compared with normal weight subjects (123.9 ± 12.2 vs. 182.4 ± 19.3 ml/h; *P *= 0.007 for group effect; *P *= 0.002 for group by time interaction, Figure 1B) and showed no differences in comparison to the obese group (150.6 ± 20.7 ml/h; *P *= 0.178 for group effect; *P *= 0.201 for group by time interaction). Additional correlation analyses revealed a positive relation between glucose infusion rates and BMI in low and normal weight subjects (*r *= 0.529; *P *= 0.043, Figure 2A and *r *= 0.587; *P *= 0.021, Figure 2B, respectively; cumulative: *r *= 0.680; *P *< 0.001), i.e. between BMI and glucose tolerance, which was found inverse in obese subjects (*r *= -0.564; *P *= 0.028; Figure 2C).

Baseline serum insulin concentrations were significantly higher in obese as compared with low and normal weight subjects (87.43 ± 8.95 vs. 42.88 ± 16.33 and 44.28 ± 12.14 pmol/l, respectively; all *P *< 0.027), while there was no difference in insulin values between the low and normal weight groups (*P *= 0.946; small insert in Figure 1A). During the hyperinsulinemic-euglycemic clamp, serum insulin concentrations increased in all groups with a significantly dampened increase in low and normal weight in comparison to obese participants (447.40 ± 42.13 and 486.55 ± 33.45 vs. 790.96 ± 74.72 pmol/l; all *P *< 0.001 for group effect; all *P *> 0.388 for group by time interaction) and comparable insulin values in the low and normal weight group (*P *= 0.594 for group effect; *P *= 0.591 for group by time interaction). Calculated ISI values were significantly higher in low and normal weight than in obese subjects (13.43 ± 1.57 and 17.82 ± 3.87 vs. 6.09 ± 1.09 mg*100*l*kg^-1^*min^-1^*mU^-1^; all *P *< 0.010) without any difference between the low and normal weight groups (*P *= 0.303).

## Discussion

Our data demonstrate that moderate underweight is associated with impaired glucose tolerance in healthy human volunteers. While a relationship between obesity and glucose intolerance is well known, our findings suggest that generally all deviations from normal BMI may be accompanied by an increased risk of developing T2DM in later life. One could thus conclude that the maintenance of body weight within the normal range has first priority in the prevention of this disease.

To our knowledge, this is the first study that shows significant associations between low weight and IGT under metabolically well controlled conditions of a standardized glucose clamp. In our study, glucose infusion rates required to maintain euglycemic glucose values were significantly decreased in low weight as compared with normal weight subjects but were comparable to those measured in obese participants. Moreover, we found a positive relation between BMI and glucose tolerance in low weight and normal weight subjects as well as a negative correlation in obese participants. To date, little is known about the relationship between low weight and IGT in healthy subjects. Former studies investigated glucose tolerance in patients with significant underweight within the scope of a pathologic state. An impairment of glucose tolerance has previously been seen in patients with cancer [4,9,10]. Additionally, IGT has also been found in middle-aged subjects with severe chronic malnutrition [3] as well as in young women with anorexia nervosa [5]. Moreover, studies in older adults indicated an association between poor nutritional status as well as hypomagnesaemia and risk of T2DM [11-13]. These findings are in line with animal studies showing that protein-energy restriction decreases glucose-induced insulin secretion and worsens post-receptor signaling of insulin [14]. Interestingly, such impairment in glucose-induced insulin release has also been seen in lean middle-aged and older patients with T2DM [15] implicating that the here described impairment of glucose tolerance in low weight young men could be a consequence of decreased insulin sensitivity in these subject. In our study, however, insulin concentrations as well as ISI were well comparable between low weight and normal weight participants indicating that BMI between 17.6 and 19.8 kg/m^2 ^is not accompanied by changes in insulin sensitivity.

## Conclusions

In summary, we found a close association between IGT and BMI equally in both low weight and obese participants. This finding is of high clinical relevance because low weight in adults has been neglected as risk factor for diabetes so far and the strong relationship suggests that the prevention of obesity and low weight may be crucial to overcome the increasing problem of T2DM. In consequence, clinicians should consider the fact that all subjects with deviations from normal BMI values, regardless of their plasma glucose levels, may display glucose intolerance and thereby could develop diabetes in later life. Thus, also subjects with moderate underweight should be advised to normalize their BMI for prevention.

## Competing interests

The authors declare that they have no competing interests.

## Authors' contributions

KMO designed the project and supervised the experiments. AS performed the experiments and acquired data. KJC and AS provided suggestions on the project, data interpretation, and the manuscript. KJC performed statistical analyses. KMO and KJC wrote the manuscript. All authors read and approved the final draft.
